# Laparoscopic Versus Open Surgery for Diverticular Disease: A Structured Review Across Clinical Settings and Operative Strategies

**DOI:** 10.1002/ags3.70285

**Published:** 2026-09-20

**Authors:** Marie Hanaoka, Yudai Fukui, Koya Hida, Hiroya Takeuchi, Yusuke Kinugasa

**Affiliations:** ^1^ Department of Gastrointestinal Surgery Institute of Science Tokyo Tokyo Japan; ^2^ Department of Gastroenterological Surgery Toranomon Hospital Tokyo Japan; ^3^ Department of Gastrointestinal Surgery Kyoto University Graduate School of Medicine Kyoto Japan; ^4^ Guideline Committee of the Japan Society for Endoscopic Surgery Tokyo Japan; ^5^ Department of Surgery Hamamatsu University School of Medicine Hamamatsu Japan

**Keywords:** colectomy, colonic diverticulitis, laparoscopy, minimally invasive surgical procedures, treatment outcome

## Abstract

Laparoscopic surgery is increasingly used to treat colonic diverticulitis; however, its role in emergency, perforated, high‐risk, and procedure‐specific settings remains uncertain. This structured narrative review evaluated and synthesized comparative evidence by clinical setting, operative strategy, and patient risk profile to provide a clinical decision‐making framework for identifying settings in which laparoscopy may offer meaningful benefits and those in which its role remains uncertain. In elective settings, randomized trials and observational studies generally demonstrated short‐term advantages of laparoscopy, including reduced postoperative morbidity, less pain, faster recovery, and shorter hospitalization, although clear mid‐ or long‐term superiority has not been established. In emergency, perforated, and high‐risk settings, observational and nationwide database studies reported favorable short‐term outcomes of laparoscopy; however, these findings were strongly influenced by patient selection, disease severity, and institutional expertise. Benefits were most consistent for resection‐based procedures but were less clear for laparoscopic Hartmann procedures and inconsistent for lavage‐ or drainage‐based strategies. Stoma creation rates tended to be lower after laparoscopy in selected cohorts, although this finding depended on disease severity and operative strategy. Evidence regarding intraoperative complications remains limited. Overall, laparoscopic surgery is associated with favorable short‐term outcomes, with the most consistent evidence arising from elective diverticular surgery. In emergency surgery and perforated disease, however, the available evidence remains predominantly observational and susceptible to selection bias. These findings support a selective, context‐dependent approach based on disease severity, patient condition, operative strategy, and institutional expertise, with careful surgical planning and timely conversion when necessary.

## Abstract 2

Laparoscopic surgery provides the most consistent short‐term benefits in elective diverticular surgery, including reduced postoperative morbidity and faster recovery. In emergency and perforated disease, evidence remains predominantly observational, and the choice of approach should be individualized according to disease severity, patient condition, operative strategy, and institutional expertise.

## 1 Introduction

Diverticular diseases are a growing global health burden, driven by aging populations and lifestyle‐related factors. Although most individuals remain asymptomatic, some develop acute diverticulitis, ranging from mild inflammation to life‐threatening perforation with generalized peritonitis. Surgical intervention is required for patients with recurrent disease, complications such as fistula or obstruction, or failure of nonoperative management. Traditionally, open colectomy has been the standard surgical approach, particularly in emergency settings and in patients with advanced or complicated diseases.

Over the past two decades, laparoscopic surgery has been increasingly used for the surgical treatment of colonic diverticulitis [1, 2, 3, 4]. Randomized controlled trials (RCTs), including the Sigma trial, have suggested short‐term benefits of elective laparoscopic sigmoidectomy over open surgery in selected patients, including reduced postoperative pain and faster recovery [5, 6]. However, these trials were limited by highly selected study populations, relatively small sample sizes, long operative times, and non‐negligible conversion rates. Moreover, mid‐ to long‐term follow‐up studies have not demonstrated clear superiority in major clinical outcomes, and economic analyses have not consistently shown a cost advantage of laparoscopy [6, 7, 8]. Taken together, randomized evidence supports certain short‐term benefits of laparoscopy but does not establish its overall superiority over open surgery.

Outside the randomized setting, large observational studies and nationwide database analyses have reported favorable associations between laparoscopic surgery and postoperative outcomes, including lower morbidity, shorter length of hospital stay, reduced stoma creation, and lower healthcare resource utilization in selected settings [1, 2, 3, 4, 9, 10, 11, 12, 13, 14, 15, 16, 17, 18, 19]. However, interpretation of these findings is complicated by the clinical heterogeneity of diverticulitis and differences in operative strategies. Outcomes may differ substantially between elective and emergency surgery, uncomplicated and perforated disease, and resection‐based procedures and non‐resection strategies such as lavage or drainage [13, 14, 15, 16, 20, 21, 22]. In addition, patient selection, disease severity, surgeon experience, institutional expertise, and conversion practices may influence both the choice of surgical approach and postoperative outcomes [11, 12, 22].

Given these uncertainties, the role of laparoscopy cannot be adequately assessed through a single clinical context or operative strategy. Thus, a structured and clinically oriented synthesis of the available evidence is needed. This review aimed to compare the outcomes of laparoscopic and open surgery for colonic diverticulitis, with particular attention paid to postoperative complications, stoma creation, and intraoperative safety. By stratifying the evidence according to clinical setting, operative strategy, and patient risk profile, we sought to provide a clinical decision‐making framework that distinguishes the effects of the operative approach from those of patient selection, disease severity, and operative strategy, rather than simply determining whether laparoscopy is uniformly superior to open surgery.

## 2 Methods

### 2.1 Study Design

**FIGURE 1:**
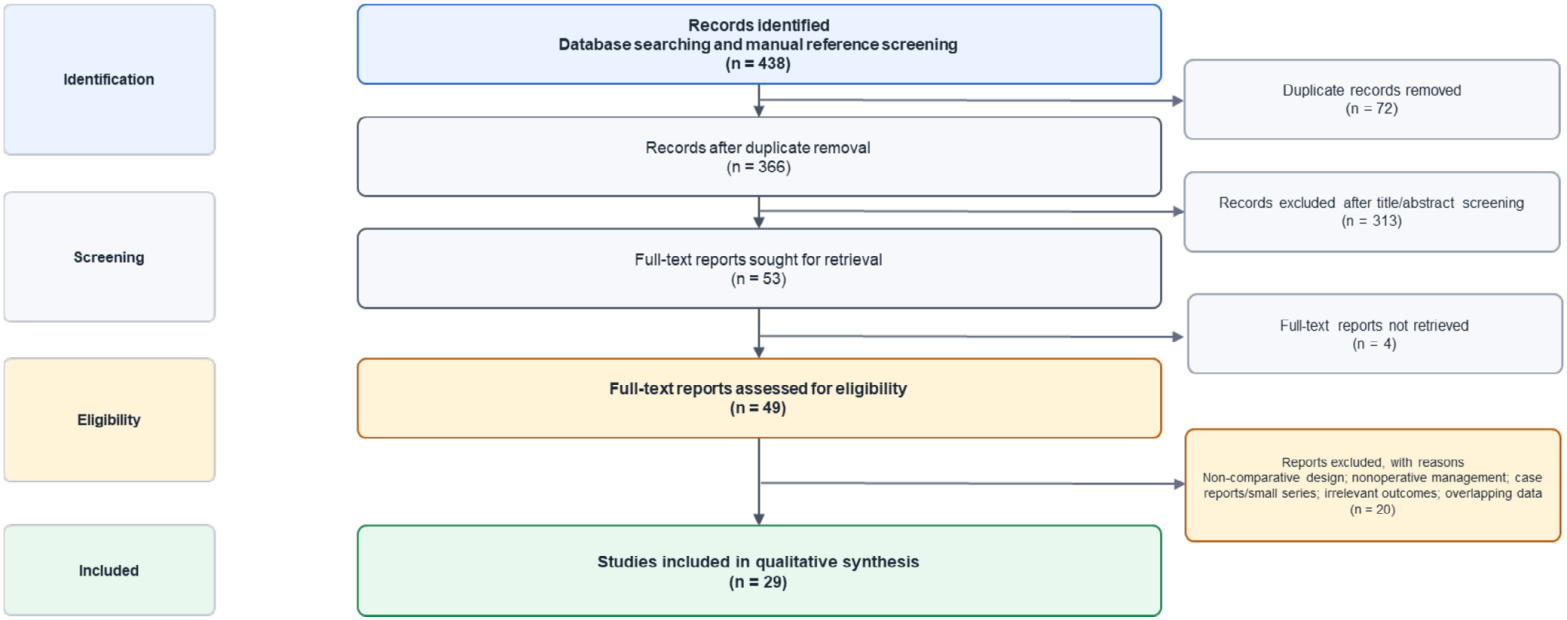
Study selection flow diagram. This flow diagram shows the process of literature identification, screening, eligibility assessment, and inclusion in this structured review. Of 438 records identified, 366 remained after duplicate removal. After title and abstract screening and full‐text assessment, 29 studies were included in the qualitative synthesis.

This structured narrative review used a predefined literature search strategy to evaluate current evidence comparing laparoscopic and open surgery for colonic diverticular disease across clinical settings and operative strategies. The study selection process is summarized in a study selection flow diagram (Figure 1).

### 2.2 Literature Search

PubMed, the Cochrane Central Register of Controlled Trials (CENTRAL) via the Cochrane Library, and Ichushi‐Web, a Japanese medical literature database, were systematically searched to identify relevant studies published between January 2010 and March 2026. The 2009 Sigma trial was also included after manual reference screening because it was a landmark randomized trial directly relevant to the review question. Additional studies were identified by manually screening the reference lists of selected articles.

### 2.3 Search Strategy

The search strategy combined terms related to diverticular disease, surgical approach, and operative procedure, including “diverticulitis,” “diverticular disease,” “laparoscopic surgery,” “minimally invasive surgery,” “open surgery,” “colectomy,” “sigmoidectomy,” and “Hartmann procedure.”

### 2.4 Eligibility Criteria

Studies were included if they met the following eligibility criteria: (1) adult patients undergoing surgery for colonic diverticular disease; (2) comparisons between laparoscopic or minimally invasive surgery (MIS) and open surgery; (3) reported clinical outcomes including mortality, morbidity, intraoperative or postoperative complications, conversion to open surgery, stoma creation, hospitalization duration, and healthcare costs.

RCTs, observational comparative studies, nationwide database studies, and meta‐analyses were considered eligible for inclusion. Meta‐analyses and systematic reviews were used to provide an overview of the evidence, but were interpreted separately from primary comparative studies to avoid double counting of overlapping populations.

Studies were excluded if they: (1) did not include a comparative open surgery group; (2) focused exclusively on non‐operative management; (3) did not report relevant clinical outcomes; (4) were case reports or small case series involving < 10 patients; or (5) focused exclusively on robotic surgery or combined robotic and laparoscopic procedures within a single MIS cohort without separate extractable laparoscopic outcomes.

### 2.5 Study Selection and Classification

Study selection and data extraction were independently performed by two reviewers, with discrepancies resolved by consensus.

Eligible studies were classified according to: (1) clinical setting, including elective surgery, emergency surgery, and perforated diverticulitis; (2) operative strategy, including resection, Hartmann procedure, and laparoscopic lavage or drainage; and (3) patient risk profile, including high‐risk populations such as patients with cirrhosis or respiratory comorbidities.

### 2.6 Data Extraction

Data on study design, data source, sample size, patient population, disease severity, surgical approach, operative strategy, and key outcomes, including mortality, postoperative morbidity, intraoperative and postoperative complications, stoma creation, conversion to open surgery, length of stay, and healthcare costs (when available) were extracted for analysis. Stoma‐related outcomes were not uniformly defined across studies and included initial stoma creation, ostomy formation during the index procedure, and subsequent stoma‐free status or survival. These endpoints were therefore interpreted separately and were not regarded as directly interchangeable. Methods used to address confounding and the principal methodological limitations of each study were also extracted.

### 2.7 Data Synthesis

Due to substantial heterogeneity in study designs, patient populations, disease severity, operative strategies, and outcome definitions among the included studies, formal quantitative meta‐analysis was not performed. Instead, the findings were qualitatively synthesized according to the clinical setting, operative strategy, and patient risk profiles.

Given the structured narrative nature of this review, no formal risk‐of‐bias assessment was performed using a standardized tool. However, the qualitative synthesis considered study design, confounding adjustment, and methodological limitations relevant to interpretation, including patient selection, disease severity, conversion practices, and institutional factors. Randomized and observational evidence was interpreted separately.

## 3 Results

Among the 29 included reports, 20 were observational studies, four reports were from randomized studies, and five were systematic reviews or meta‐analyses. Randomized evidence was confined primarily to elective surgery, whereas evidence for emergency surgery, perforated disease, and high‐risk populations was predominantly observational. Although several observational studies used multivariable or propensity‐score adjustment, residual confounding by indication, disease severity, patient fitness, operative strategy, and institutional expertise remained likely. Additional contextual references addressing ureteral injury and fluorescence‐guided ureter visualization, including publications outside the predefined search period, were cited to support the discussion of intraoperative safety but were not included in the main comparative synthesis.

The study selection process is summarized in Figure 1. The study characteristics are summarized in Table 1. Key quantitative findings according to clinical setting and operative strategy are summarized in Table 2, and additional outcomes and detailed findings are presented in Table S1.

**TABLE 1:** Studies comparing laparoscopic and open surgery for diverticular disease.

Ref	Study (first author, year)	Country/region	Design	Setting/data source	*N*	Population/Hinchey classification (as reported)	Surgical comparison/procedure	Key outcomes	Main direction/interpretation	Confounding adjustment/methodological safeguards	Principal methodological limitation/risk of bias
[1]	Masoomi et al. (2011)	USA	Retrospective database	NIS/national	1 24 734	Elective surgery for diverticulitis	LS versus OS/colectomy	Morbidity, mortality, LOS, costs	Favors LS for short‐term outcomes	Multivariable risk‐adjusted analysis of NIS data	Retrospective administrative data; selection and residual confounding; limited disease‐severity, conversion, surgeon, and long‐term information
[2]	Kakarla et al. (2012)	USA	Retrospective database	ACS‐NSQIP/national	7629	Elective colectomy for symptomatic diverticulosis/diverticular disease	LS versus OS/colectomy	Morbidity, LOS	Favors LS in elective surgery	Multivariable risk‐adjusted analysis of ACS‐NSQIP data	Retrospective database study; baseline imbalance and residual confounding; limited disease‐severity, conversion, and long‐term data
[3]	Siddiqui et al. (2010)	International/multiple	Meta‐analysis	Elective studies including the Sigma trial	918	Elective sigmoid colectomy for diverticular disease	LS versus OS/sigmoidectomy	Complications, recovery, operative time, blood loss	Favors LS for recovery/complications; longer operative time	Study‐level meta‐analysis of comparative studies with pooled estimates	Predominantly nonrandomized studies; clinical/statistical heterogeneity and selection bias; overlap with primary studies in this review
[4]	Wu et al. (2017)	International/multiple	Systematic review and meta‐analysis	Multiple studies	28 studies; total N not available	Diverticulitis across elective and emergency/complicated settings; study‐specific severity definitions	LS versus OS	Overall perioperative outcomes	Generally favors LS	Systematic review and meta‐analysis with subgroup and sensitivity analyses	Predominantly retrospective evidence; marked heterogeneity and confounding in source studies; pooled associations may overstate causality
[5]	Klarenbeek et al. (2009)	Netherlands/Europe	RCT	Sigma trial	104	Symptomatic diverticulitis requiring elective sigmoid resection	LS versus OS/sigmoid resection	Major morbidity, postoperative complications, pain, recovery, hospital stay	LS sigmoid resection reduced major morbidity and improved short‐term recovery compared with OS surgery	Random allocation in the multicenter Sigma trial	Small, selected elective cohort; 19.2% conversion and short follow‐up; limited generalizability
[6]	Klarenbeek et al. (2011)	Netherlands/Europe	RCT follow‐up	Sigma trial follow‐up	104	Elective diverticular disease	LS versus OS/sigmoidectomy	Morbidity, QoL, GI function, long‐term outcomes	Early benefit; no clear long‐term superiority	Randomization inherited from the Sigma trial; follow‐up analysis	Secondary report of the same small Sigma cohort; attrition and limited power for long‐term outcomes
[7]	Raue et al. (2011)	Germany	RCT	Multicenter	149	Symptomatic sigmoid diverticular disease; Stock/Hansen Stage II–III	LS versus OS/sigmoidectomy	Midterm morbidity and clinical outcomes	No clear midterm advantage for LS	Random allocation in a prospective multicenter trial	Recruitment stopped before the target sample size; likely underpowered; selected elective population
[8]	Klarenbeek et al. (2011)	Netherlands/Europe	RCT follow‐up	Sigma trial economic analysis	104	Elective symptomatic diverticular disease	LS versus OS/sigmoidectomy	Operative cost, total healthcare cost	Higher operative cost but comparable overall healthcare cost	Randomization inherited from the Sigma trial; trial‐based economic analysis	Secondary economic analysis of the same small Sigma cohort; context‐specific costs and limited generalizability
[9]	Le et al. (2024)	USA	Retrospective database	ACS‐NSQIP/national	9194	Emergent colectomy for diverticulitis	MIS versus planned OS/colectomy	Major adverse events, stoma, LOS, discharge	Favors MIS in selected emergency cases	Multivariable regression adjustment of ACS‐NSQIP data	Retrospective 30‐day database study; confounding by indication and patient fitness; conversion and procedure‐selection bias
[10]	Hajirawala et al. (2022)	USA	Retrospective database	ACS‐NSQIP/national	3487	Urgent colectomy for acute diverticulitis	LS versus OS/urgent colectomy	Mortality, complications, ileus, LOS	Mortality/overall complications similar; ileus and LOS favor LS	Univariable and multivariable regression analyses	Retrospective ACS‐NSQIP data; residual confounding by urgency/severity and surgeon selection; 30‐day outcomes only
[11]	Letarte et al. (2015)	Canada	Retrospective comparative study	Emergency admission/single or multicenter cohort	125	Complicated diverticular disease requiring emergency admission	LS versus OS/colonic resection	Morbidity, oral intake, LOS	Favors LS in selected complicated emergency cases	Retrospective comparative analysis; limited covariate adjustment	Small selected emergency cohort; confounding by indication, center expertise, and disease severity; limited power
[12]	Ebrahimian et al. (2023)	USA	Retrospective national analysis	National database	1,10 281	Elective colectomy for diverticulitis; disease severity not reported	MIS (including converted cases) versus planned OS/colectomy; hospital‐volume strata	Conversion‐to‐OS, mortality, hospital volume	Higher MIS volume was associated with lower conversion; converted MIS was not associated with worse outcomes than planned OS surgery in selected cohorts	Multivariable national database analysis with hospital‐volume stratification	Administrative data; residual confounding by case complexity and surgeon skill; conversion is not randomized and coding may be imperfect
[13]	Vennix et al. (2016)	Netherlands/international collaboration	PSM cohort	Multicenter	117	Perforated diverticulitis with purulent or fecal peritonitis (Hinchey III–IV)	LS versus OS/acute sigmoidectomy	Morbidity, LOS, operative time, blood loss, intraoperative complications	Favors LS for selected short‐term outcomes; longer operative time	PSM on measured clinical and operative factors	Small matched cohort; unmeasured confounding and temporal/center effects remain; selected LS candidates
[14]	Lee et al. (2020)	USA	Retrospective comparative study	Institutional/database cohort	3756	Perforated diverticulitis requiring emergent sigmoid resection	LS versus OS/emergent sigmoid resection	Morbidity, LOS, stoma‐free outcomes	Favors LS in selected perforated cases	PS weighting; conversion examined separately and by intention‐to‐treat	Retrospective ACS‐NSQIP study; residual confounding, 30‐day follow‐up, and sensitivity to conversion classification
[15]	Moghadamyeghaneh et al. (2021)	USA	Retrospective database	NSQIP/national	2937	Perforated diverticulitis	MIS versus OS/colectomy	Morbidity, LOS, respiratory complications, conversion	Favors MIS in selected patients despite conversion risk	Multivariable logistic regression adjustment	Retrospective NSQIP study; high conversion rate, selection bias, limited physiologic/severity detail, and 30‐day outcomes
[16]	Paasch et al. (2023)	Germany	Retrospective observational study	Single center consecutive cohort	77	Perforated diverticulitis	LS versus OS/resection	Feasibility, morbidity, LOS	Supports feasibility of LS resection in selected patients	Consecutive retrospective cohort; no robust adjusted comparison reported	Very small single‐center cohort; strong selection and expertise bias; limited power and generalizability
[20]	Amodu et al. (2024)	USA	PSM database study	ACS‐NSQIP/national	1845	Complicated diverticulitis undergoing Hartmann procedure	LS versus OS/Hartmann procedure	SSI, mortality, major morbidity, LOS	Superficial SSI lower; no consistent advantage for major outcomes	PSM of ACS‐NSQIP patients	Residual unmeasured confounding; limited severity and intraoperative detail; 30‐day database outcomes
[21]	Turley et al. (2013)	USA	PSM database study	National/institutional database	1145	Emergency diverticulitis undergoing Hartmann procedure	LS versus OS/Hartmann procedure	Mortality, morbidity, LOS	No clear mortality or morbidity advantage	PSM analysis	Retrospective database study; residual confounding, small LS sample, and limited severity/technical detail
[22]	Lin et al. (2020)	International/multiple	Systematic review and meta‐analysis	Emergency LS studies	918	Emergency complicated diverticulitis; Hinchey grade varied across included studies (II–IV)	LS resection or lavage/drainage versus OS resection	Morbidity, severe complications	LS resection may reduce morbidity; LS lavage/drainage less favorable for severe complications	Systematic review/meta‐analysis with study‐level quality assessment	Heterogeneous procedures and patient severity; mainly observational studies, selection bias, and indirect comparisons
[17]	Kazi et al. (2020)	USA	Retrospective database	National database	1145	Acute colonic diverticulitis with cirrhosis	LS versus OS/colectomy	Mortality, LOS, costs	Favors LS in selected cirrhotic patients	Multivariable regression adjustment of national database data	Retrospective administrative data; coding and selection bias; limited cirrhosis and diverticulitis severity detail
[18]	Patel et al. (2020)	USA	PSM database study	National data 2005–2017	70 420	Diverticulitis with preoperative respiratory comorbidity	LS versus OS/colectomy	Mortality, morbidity, wound complications, reoperation, LOS	Favors LS in patients with respiratory comorbidity	PSM on demographic and preoperative risk factors	Observational data; fitter patients may have been selected for LS; residual physiologic/severity confounding and coding limitations
[19]	Braschi et al. (2022)	USA	Retrospective national database study	National database	15 316	Elderly patients with diverticulitis undergoing elective or urgent/emergent surgery; disease severity not reported	LS versus OS	30‐day morbidity, SSI, LOS	Favors LS in selected elderly patients	CEM; elective and non‐elective cohorts analyzed separately	Retrospective matched data; residual frailty/severity confounding; short‐term outcomes and limited generalizability
[23]	Mbadiwe et al. (2013)	USA	Retrospective database	ACS‐NSQIP/national	11 981	Complicated diverticulitis undergoing colectomy	LS versus OS/colectomy; primary anastomosis or colostomy subgroups	Postoperative morbidity, mortality, and subgroup outcomes by primary anastomosis versus colostomy	Favors LS, particularly for lower morbidity in patients undergoing primary anastomosis	Multivariable risk‐adjusted ACS‐NSQIP analysis with procedure subgroups	Retrospective database study; confounding by operative strategy and disease severity; 30‐day outcomes and limited technical detail
[24]	Zimniak et al. (2025)	Germany	Multicenter retrospective study	Multicenter	101	Recurrent sigmoid diverticulitis with fistula (complicated/fistulizing disease)	LS versus OS	LOS, bowel recovery, ICU stay, drain removal	Favors LS for recovery outcomes	Multicenter retrospective analysis with multivariable logistic regression	Small fistula cohort; residual selection and center‐expertise bias; heterogeneous anatomy and operative complexity
[25]	Trejo‐Avila et al. (2021)	International/multiple	Systematic review and meta‐analysis	Colovesical fistula studies	227	Diverticular colovesical fistula (complicated/fistulizing disease)	LS versus OS	Complications, LOS, leak, SSI, stoma, mortality	Favors LS for total complications and LOS; other outcomes comparable	Random‐ or fixed‐effects meta‐analysis of comparative observational studies	Small, heterogeneous nonrandomized studies; selection bias, variable fistula definitions, and limited power for rare outcomes
[26]	Kosuge et al. (2024)	Japan	Retrospective comparative study	Japanese institutional cohort	16	Sigmoid colovesical fistula caused by diverticulitis (complicated/fistulizing disease)	LS versus OS	Blood loss, LOS, surgical outcomes	Suggests lower blood loss and shorter hospitalization with LS	Small retrospective comparison; no multivariable adjustment reported	Only 16 patients; substantial selection bias, baseline imbalance, and imprecision; single‐center Japanese cohort
[27]	Horsey et al., (2022)	USA	Retrospective database	ACS‐NSQIP/national	484	Right‐sided colonic diverticulitis requiring resection	MIS versus OS/resection	Morbidity, LOS	Comparable morbidity and shorter LOS with MIS.	Multivariable risk‐adjusted ACS‐NSQIP analysis	Retrospective 30‐day database study; limited right‐sided disease detail, residual confounding, and possible MIS coding heterogeneity
[28]	Kwon et al. (2012)	Korea	Retrospective comparative study	Two‐center	59	Complicated right colonic diverticulitis	LS versus OS	Complications, feasibility	Comparable outcomes; supports feasibility of LS in selected cases	Retrospective two‐center comparison; no robust adjustment reported	Small cohort (*n* = 59); selection and center bias, limited power, and uncertain generalizability
[29]	Abraha et al. (2017)	International/multiple	Cochrane systematic review of RCTs	Randomized evidence	392	Patients undergoing sigmoid resection in three RCTs; heterogeneous eligibility including Hinchey I–II and Stock/Hansen Stage II–III	LS versus OS/resection	Pain, morbidity, operative time, safety	LS may reduce pain but requires longer operative time.	Cochrane risk‐of‐bias and certainty assessment of randomized trials	Only three small RCTs; low‐certainty evidence due to bias, attrition, imprecision, and lack of blinded outcome assessment

*Note:* Hinchey classification is shown only when explicitly reported in the original study. When Hinchey classification is not reported, disease severity or clinical setting is summarized using the terminology of the original study. If neither is available, “Not reported” is shown.

Abbreviations: ACS‐NSQIP, American College of Surgeons National Surgical Quality Improvement Program; CEM, coarsened exact matching; GI, gastrointestinal; ICU, intensive care unit; LOS, length of stay; LS, laparoscopic surgery; MIS, minimally invasive surgery; NIS, Nationwide Inpatient Sample; NR, not reported; OS, open surgery; PS, propensity score; PSM, propensity score matching; QoL, quality of life; RCT, randomized controlled trial; SSI, surgical site infection.

**TABLE 2:** Summary of evidence by clinical domain.

Evidence domain	Refs	Study design/data source	Postoperative morbidity/complications	Numeric details/representative findings	Mortality	Costs/charges	Key outcome summary	Overall direction	Evidence interpretation/principal limitation
Elective surgery	[3, 5, 6, 7, 8, 29]	Randomized controlled trials, meta‐analysis, and systematic review	No definitive pooled reduction in complications was established	Three RCTs; *n* = 392 overall; no definitive reduction in complications was established	Not reported	Higher operative costs with laparoscopy	Short‐term recovery favors laparoscopy, but operative costs are higher; mid‐ or long‐term superiority was not established	Favors laparoscopy for short‐term recovery only	Randomized evidence supports short‐term recovery benefits, but the trials were small, involved selected patients, and were underpowered for uncommon or long‐term outcomes
Elective/mixed surgery	[1, 2, 3, 4, 12, 23]	Large database studies and meta‐analytic studies	Lower overall morbidity with laparoscopy, including in complicated diverticulitis	Complicated diverticulitis cohort: *n* = 11 981; the primary‐anastomosis subgroup favored laparoscopy	Not consistently reported	Lower hospital charges/costs with laparoscopy	Lower LOS, lower hospital costs, and fewer wound complications with laparoscopy	Favors laparoscopy	Large samples improve precision, but the predominantly observational findings remain susceptible to selection bias, residual confounding, and dataset overlap
Emergency surgery	[9, 10, 11, 23]	Observational database cohort	Lower MAE with MIS	42.0% versus 56.4%; adjusted MAE AOR 0.56 (95% CI 0.50–0.63)	Lower mortality with laparoscopy: 4.1% versus 8.8%	Not reported	Lower MAE, ileus, ostomy formation, mortality, LOS, and postoperative morbidity with MIS/laparoscopy, particularly in selected patients with complicated diverticulitis undergoing primary anastomosis	Favors MIS/laparoscopy in selected emergency cases	Predominantly retrospective evidence; confounding by indication, physiologic status, disease severity, and surgeon selection limits causal interpretation
Conversion‐related outcomes	[9, 12]	Observational cohorts	Lower MAE after converted MIS versus planned open surgery	AOR 0.78 (95% CI 0.65–0.93)	Lower mortality was observed after conversion to open surgery than after planned open surgery: AOR 0.30 (95% CI 0.10–0.60)	Not reported	In selected observational cohorts, converted MIS was associated with lower rates of nonhome discharge, MAE, ostomy formation, and mortality	Conversion was not necessarily associated with worse outcomes than planned open surgery in selected observational cohorts	Conversion analyses are observational and sensitive to patient selection, intraoperative difficulty, institutional volume, and conversion classification
Perforated diverticulitis: laparoscopic resection	[13, 14, 15, 16]	Propensity score–matched cohort	Lower morbidity with laparoscopy	Overall morbidity: 43.6% versus 66.2%; severe morbidity: 12.8% versus 19.5%	Mortality was similar or numerically lower with laparoscopy: 2.6% versus 3.9%	Not reported	Lower LOS, morbidity, severe morbidity, and wound infection and a higher stoma‐free probability with laparoscopy	Favors laparoscopic resection in selected perforated cases	Propensity methods reduce measured imbalance but cannot fully address unmeasured severity, physiology, contamination, or surgeon‐expertise differences
Hartmann procedure	[20, 21]	Unadjusted cohort and NSQIP propensity score–matched cohort	Adjusted analyses did not confirm lower morbidity or mortality with laparoscopy	Not reported	Adjusted analyses did not confirm lower mortality with laparoscopy	Not reported	Shorter LOS and lower superficial SSI rates with laparoscopic Hartmann procedure	Limited advantage	Matched database studies remain vulnerable to residual confounding and limited severity detail; evidence supports only limited short‐term advantages
Lavage/drainage strategy	[22]	Systematic review/meta‐analysis	Laparoscopic lavage/drainage did not show a clear safety advantage; laparoscopic colon resection showed more favorable morbidity than lavage/drainage in comparative evidence	Not reported	Not applicable to this row	Not reported	Laparoscopic lavage/drainage showed no clear safety advantage; severe complications may be more frequent than with open resection	Does not favor lavage/drainage	Evidence is heterogeneous and partly indirect because lavage/drainage and resection are used in different patients and are not equivalent strategies
High‐risk populations	[17, 18, 19]	Observational cohorts	Lower 30‐day morbidity with laparoscopy	Elective surgery: OR 0.47; non‐elective surgery: OR 0.76	Lower mortality with laparoscopy in the cirrhosis cohort	Lower costs with laparoscopy in cirrhosis cohort	Lower morbidity, SSI, LOS, mortality, and costs with laparoscopy in selected high‐risk cohorts	Observational associations favor laparoscopy; causal certainty is low	Entirely observational evidence; preferential selection of physiologically fitter patients for laparoscopy may substantially influence results
Fistulizing disease	[24, 25, 26]	Multicenter cohort, meta‐analysis, and smaller observational cohort	Fewer postoperative complications with laparoscopy	CVF meta‐analysis: OR 0.55 (95% CI 0.30–0.99); Japanese cohort: 1/7 versus 5/9, *p* = 0.09	No clear mortality difference: OR 0.18	Not reported	Shorter LOS and fewer overall postoperative complications with laparoscopy in selected fistula/CVF cohorts	Favors laparoscopy in selected fistula cases	Evidence is based on small retrospective cohorts and a meta‐analysis of nonrandomized studies; anatomy, complexity, and selection vary across studies
Disease location	[27, 28]	NSQIP cohort and smaller observational cohorts	Comparable or lower short‐term morbidity with MIS	Not reported	Not reported	Not reported	Shorter hospital stay and comparable or lower short‐term morbidity with MIS	MIS feasible; may improve short‐term recovery	Limited retrospective evidence with small right‐sided cohorts; residual confounding and restricted generalizability warrant cautious interpretation

*Note:* OS indicates open surgery in this table, not overall survival. “Not reported” indicates that the outcome was not reported in the cited evidence; “Not consistently reported” indicates that the outcome was reported heterogeneously or was not consistently extractable across studies. Stoma‐related outcomes were not uniformly defined across studies and included stoma or ostomy formation at the index operation and subsequent stoma‐free status or survival. These outcomes were interpreted as distinct endpoints and were not regarded as directly interchangeable.

Abbreviations: AOR, adjusted odds ratio; CI, confidence interval; COPD, chronic obstructive pulmonary disease; CVF, colovesical fistula; ICU, intensive care unit; LCR, laparoscopic colon resection; LLD, laparoscopic lavage/drainage; LOS, length of stay; LS, laparoscopic surgery; MAE, major adverse events; MD, mean difference; MIS, minimally invasive surgery; NIS, National Inpatient Sample; NSQIP, National Surgical Quality Improvement Program; OCR, open colon resection; OR, odds ratio; OS, open surgery; PS, propensity score; PSM, propensity score matching; RCT, randomized controlled trial; SBF, sigmoid‐bladder fistula; SSI, surgical site infection; SVF, sigmoid‐vaginal fistula.

### 3.1 Elective Diverticular Disease

In the elective setting, evidence from RCTs, meta‐analyses, and large observational studies generally supports short‐term benefits of laparoscopic surgery compared with open surgery. Large database studies have reported lower postoperative morbidity, fewer wound complications, shorter hospital stays, and lower hospital costs after laparoscopic colectomy [1, 2]. Meta‐analyses have likewise demonstrated reduced overall complications and faster recovery with laparoscopy, although operative times were generally longer [3, 4]. The results of RCTs supported these short‐term benefits, including reduced postoperative ileus, shorter hospitalization, and less early postoperative pain after laparoscopic sigmoidectomy [5, 6]. However, mid‐ and long‐term follow‐up studies have shown no clear differences in mortality, morbidity, gastrointestinal function, or quality of life between laparoscopic and open surgery [6, 7]. Cost analyses from the Sigma trial also suggested comparable overall healthcare costs despite higher operative costs associated with laparoscopy [8].

### 3.2 Emergency and Perforated Diverticulitis

In selected emergency settings, observational studies and national database analyses have reported favorable short‐term outcomes with laparoscopic or minimally invasive surgery, including fewer major adverse events, lower ostomy formation rates, lower ileus rates, shorter hospital stay, and lower nonhome discharge rates compared with open surgery [9, 10, 11]. In one large emergency colectomy cohort, MIS was associated with lower major adverse events than open surgery (42.0% vs. 56.4%; adjusted odds ratio 0.56, 95% CI 0.50–0.63), as well as lower 30‐day mortality (4.1% vs. 8.8%), lower ostomy formation (35.8% vs. 84.8%), lower ileus rates (24.4% vs. 35.4%), and shorter hospital stay (median 7 vs. 9 days; all *p* < 0.001) [9]. Observational analyses of conversion found that outcomes after conversion from MIS to open surgery were not necessarily worse than those after planned open surgery in selected cohorts [9, 12]. Importantly, institutional experience appears to be an important modifier of these outcomes, as higher hospital MIS volumes are associated with lower conversion‐to‐open rates in patients undergoing colectomy for diverticulitis [12].

Evidence in perforated diverticulitis was predominantly observational and heterogeneous. Studies evaluating laparoscopic resection have reported lower morbidity, shorter hospital stay, and higher stoma‐free survival compared with open sigmoidectomy [13, 14]. In a propensity score‐matched cohort, laparoscopic sigmoidectomy was associated with lower overall morbidity than open surgery (44% vs. 66%, *p* = 0.016), shorter hospital stay (7 vs. 9 days, *p* = 0.016), fewer wound infections (3% vs. 29%, *p* = 0.009), and a higher 12‐month probability of being stoma‐free among patients undergoing Hartmann's procedure (0.88 vs. 0.64, *p* = 0.019) [13]. Similarly, large database studies have suggested lower morbidity, shorter hospitalization, and fewer respiratory complications after laparoscopic surgery despite high conversion rates [15, 16]. An American College of Surgeons National Surgical Quality Improvement Program (ACS‐NSQIP)‐based study of complicated diverticulitis also reported lower postoperative morbidity for laparoscopy, particularly among patients undergoing primary anastomosis [23]. However, outcomes vary according to operative strategy. Laparoscopic Hartmann procedures have shown limited advantages, such as reduced superficial surgical site infections (SSIs), but no consistent reduction in mortality, hospital stay, or major complications [20, 21]. Additionally, laparoscopic lavage or drainage is not equivalent to laparoscopic resection and is associated with unfavorable or inconsistent outcomes compared with open resection‐based strategies [22].

### 3.3 High‐Risk Populations

Several observational studies suggested that laparoscopic surgery may be beneficial in selected high‐risk populations. In patients with cirrhosis, laparoscopy is associated with lower mortality, shorter length of stay, and lower costs than open colectomy [17]. In patients with respiratory comorbidities, laparoscopy is associated with lower mortality, morbidity, wound complications, reoperation, and shorter length of stay [18]. Length of stay was shorter with laparoscopy among smokers (5.3 vs. 9.5 days), patients with dyspnea (6.8 vs. 11.1 days), and patients with chronic obstructive pulmonary disease (7.4 vs. 12.6 days, all *p* < 0.0001) [18]. In elderly patients, laparoscopic surgery was associated with lower 30‐day morbidity in both elective and non‐elective settings (OR 0.47, 95% CI 0.38–0.58; and OR 0.76, 95% CI 0.58–0.98, respectively), fewer SSIs (OR 0.43, 95% CI 0.33–0.57; and OR 0.66, 95% CI 0.44–0.98, respectively), and shorter hospital stay (mean difference 1.7 days, 95% CI 1.5–1.9; and 1.2 days, 95% CI 0.43–2.1, respectively) [19]. However, all evidence in these high‐risk populations was observational.

### 3.4 Procedure‐Specific Disease

For diverticular fistulas, including colovesical and colovaginal fistulas, laparoscopic surgery has been associated with shorter hospital stay, faster bowel recovery, shorter ICU stay, and earlier drain removal compared with open surgery [24]. A meta‐analysis of diverticular colovesical fistulas also showed lower total postoperative complications with laparoscopy (OR 0.55, 95% CI 0.30–0.99) and shorter length of stay (mean difference −2.89 days, 95% CI −4.20 to −1.58), while mortality, anastomotic leak, SSI, and stoma rates were comparable [25]. Similarly, a small comparative study suggested reduced blood loss (*p* < 0.01) and shorter hospitalization (*p* = 0.04) after laparoscopic surgery [26].

For right‐sided diverticulitis, laparoscopic or minimally invasive resection was associated with lower morbidity and shorter length of hospital stay in NSQIP data [27]. Smaller comparative studies on complicated right‐sided diverticulitis reported comparable outcomes between laparoscopic and open surgeries, supporting its feasibility in selected patients [28].

### 3.5 Operative Parameters and Intraoperative Outcomes

Operative time, blood loss, and intraoperative complications have shown less consistent differences between laparoscopic and open surgery than postoperative morbidity and hospitalization duration. In elective diverticular disease, evidence from meta‐analyses showed that laparoscopic sigmoid resection was associated with a longer operative time than open surgery, whereas the estimated blood loss was lower in the laparoscopic group [3]. A Cochrane review of randomized evidence similarly reported longer operative time with laparoscopic surgery (mean difference + 49.28 min), although the certainty of the evidence was low [29]. In perforated diverticulitis, a propensity score‐matched cohort study showed a longer operative time with laparoscopic sigmoidectomy than with open surgery (127 vs. 96.5 min), while intraoperative complications were uncommon in both groups (1 vs. 2 cases), and intraoperative blood loss < 100 mL was numerically more frequent with laparoscopy but not statistically significant (74% vs. 42%, *p* = 0.117) [13]. Smaller studies on diverticular fistulas also suggested reduced blood loss with laparoscopic surgery, including a comparative study of sigmoid colovesical fistula reporting significantly lower blood loss after laparoscopy (*p* < 0.01) [26]. Overall, current evidence is insufficient to conclude that laparoscopy reduces intraoperative complications. Rather, laparoscopic surgery appears to be associated with longer operative times, whereas reduced blood loss may be observed in selected settings.

### 3.6 Summary of Findings

Among the outcomes evaluated, postoperative complications, stoma creation, and intraoperative safety are particularly important clinical endpoints. Across the available literature, laparoscopic surgery has generally been associated with fewer postoperative complications and faster recovery than open surgery in elective and selected emergency settings [1, 2, 3, 4, 5, 6, 7, 8, 9, 10, 11], with similar favorable trends reported in selected high‐risk populations [17, 18, 19]. Stoma creation rates also tended to be lower after laparoscopy in selected resection‐based cohorts; however, this finding was strongly influenced by disease severity, patient selection, and operative strategy, which may be particularly important in emergency settings [9, 10, 11, 12, 13, 14, 15, 16, 23].

**FIGURE 2:**
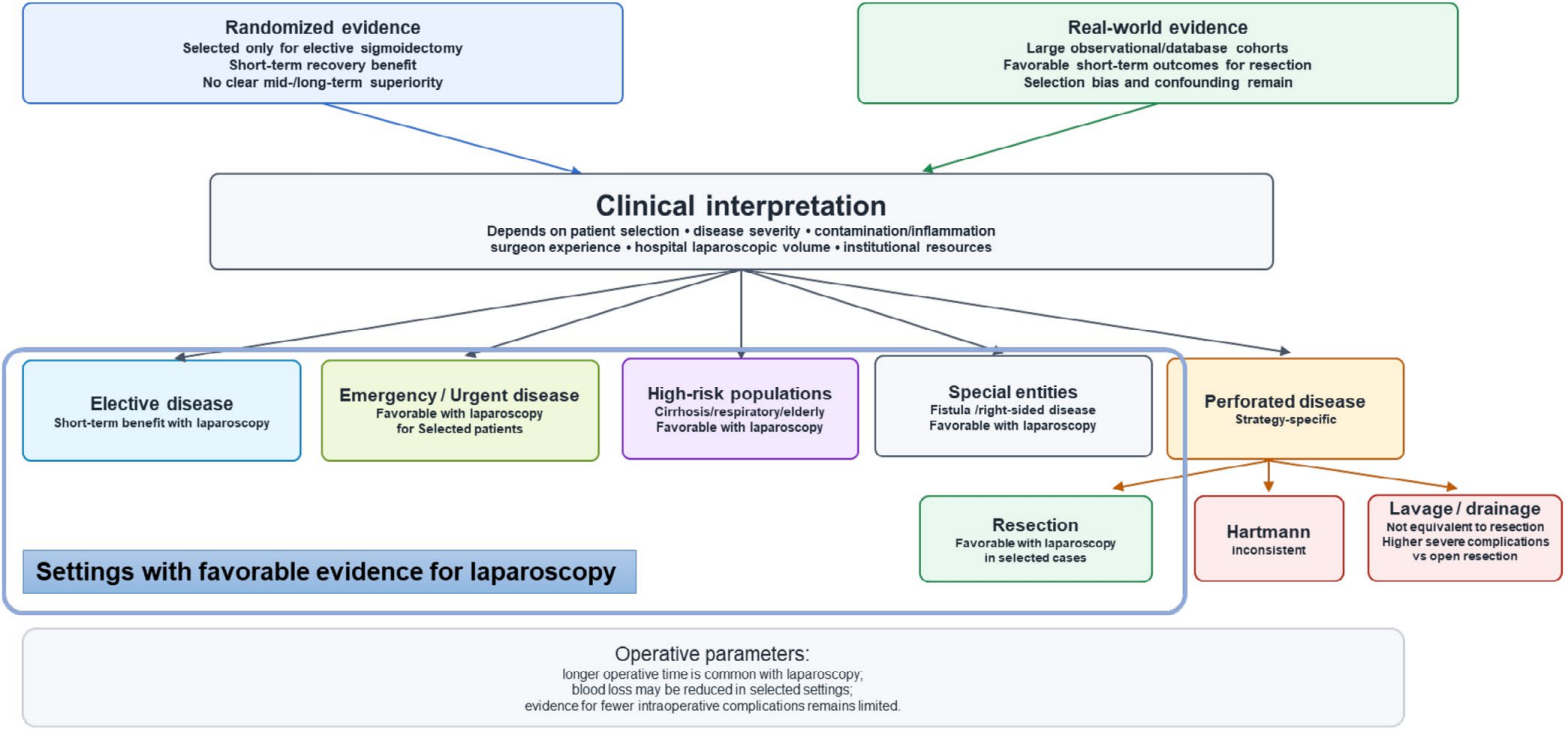
Clinical framework for interpreting laparoscopic versus open surgery for diverticular disease. Evidence favoring laparoscopy was mainly observed in elective and selected emergency settings and in high‐risk, fistulizing, and right‐sided diverticular disease. Outcomes of perforated disease were strategy‐specific; laparoscopic resection may be favorable in selected cases, whereas Hartmann procedures yielded inconsistent results, and lavage or drainage was not equivalent to resection.

In contrast, evidence regarding intraoperative complications remains limited. Intraoperative adverse events have been inconsistently reported and are rarely analyzed as independent outcomes [3, 13, 26, 29]. Therefore, current evidence supports laparoscopy primarily for reducing postoperative morbidity and facilitating recovery, particularly in elective and selected resection‐based settings. However, outcomes in emergency surgery and perforated disease are strongly influenced by patient selection and operative strategy, and the effects of laparoscopy on stoma creation and intraoperative complications remain uncertain (Figure 2).

## 4 Discussion

Laparoscopic surgery is an increasingly important approach for the management of diverticular diseases. The results of this review demonstrated its consistent short‐term benefits in elective settings and potential usefulness in selected emergency, perforated, high‐risk, and procedure‐specific settings. However, the effectiveness of laparoscopy varies according to the clinical context, disease severity, operative strategy, patient risk profile, and institutional experience. Robot‐assisted colectomy has also emerged as a minimally invasive approach and is increasingly used in colorectal surgery. However, robot‐assisted surgery was intentionally excluded from the present review, which focused specifically on evidence comparing laparoscopic surgery with open surgery.

Across both elective and emergency settings, the available evidence indicates that the primary advantage of laparoscopic surgery is improved short‐term postoperative recovery, rather than long‐term superiority. Large database studies, meta‐analyses, and RCTs have demonstrated reduced postoperative morbidity, wound complications, ileus, and hospitalization, pain, and hospital costs with laparoscopy compared with open surgery [1, 2, 3, 4, 5, 6]. However, mid‐ and long‐term follow‐up studies have not demonstrated a clear superiority in terms of mortality, morbidity, gastrointestinal function, quality of life, or overall satisfaction [6, 7]. Observational studies have also suggested that laparoscopy may be associated with improved short‐term outcomes in selected patients undergoing emergency surgery for complicated diverticulitis. However, these findings should be interpreted cautiously because patient selection, disease severity, operative strategy, and institutional factors strongly influence both the choice of surgical approach and postoperative outcomes. In emergency surgery, rapid and reliable source control should take priority over completion of a minimally invasive procedure. Conversion to open surgery should therefore be regarded as an appropriate intraoperative decision made for patient safety rather than a technical failure.

In perforated diverticulitis, the benefits of laparoscopy appear to depend on the operative strategy. Laparoscopic resection has been associated with reduced morbidity, shorter hospital stay, and higher stoma‐free survival in selected cohorts [13, 14, 15, 16, 23]. In contrast, laparoscopic Hartmann procedures have shown limited advantages, with no consistent reduction in mortality, major complications, or length of stay [20, 21]. Laparoscopic lavage or drainage should not be interpreted as equivalent to laparoscopic resection, as lavage‐ or drainage‐based strategies have shown unfavorable or inconsistent outcomes compared with resection‐based approaches [22]. Accordingly, the favorable evidence for laparoscopy in perforated diverticulitis should be interpreted as applying primarily to selected patients undergoing laparoscopic resection rather than uniformly to Hartmann procedures or lavage‐ or drainage‐based strategies. Primary anastomosis, Hartmann's procedure, and lavage or drainage are generally selected for clinically different patient populations according to disease severity, patient physiology, and operative indications. Therefore, the available observational evidence cannot reliably distinguish the effect of the laparoscopic approach itself from those of patient selection and operative‐strategy selection.

The potential benefits of laparoscopy in high‐risk populations are noteworthy. Studies of patients with cirrhosis, respiratory comorbidities, and advanced age have suggested that laparoscopy may reduce morbidity, mortality, length of hospital stay, and healthcare costs in selected patients [17, 18, 19]. Similarly, procedure‐specific studies, including those on diverticular fistulas and right‐sided diverticulitis, have suggested improved short‐term recovery or comparable outcomes when laparoscopic surgery is technically feasible [24, 25, 26, 27, 28]. However, these findings are derived predominantly from observational evidence, and preferential selection of physiologically fitter patients for laparoscopy may have contributed substantially to the reported associations.

Intraoperative safety outcomes have been reported less consistently. Although reduced ureteral injury during laparoscopic surgery has recently attracted attention, diverticulitis‐specific comparative evidence remains limited, and the available data are insufficient to determine whether laparoscopy increases or decreases this risk. Fluorescence‐guided techniques may improve ureter visualization; however, their ability to prevent ureteral injury remains uncertain [30, 31, 32, 33].

A key challenge in interpreting the available evidence is integrating RCT data with real‐world observational evidence in diverticulitis, a clinically heterogeneous disease ranging from elective surgery for recurrent disease to emergency surgery for perforation or generalized peritonitis. RCTs provide the most reliable evidence but have mainly evaluated elective sigmoidectomy in selected patients and are limited by small sample sizes, heterogeneous inclusion criteria, and enrollment challenges [5, 6, 7, 29]. Conversely, large observational studies and database analyses include broader clinical settings and generally favor laparoscopy, but are susceptible to selection bias, residual confounding, coding errors, and differences in surgeon or institutional expertise [1, 2, 3, 4, 9, 10, 11, 12, 13, 14, 15, 16, 17, 18, 19, 23]. Therefore, real‐world evidence should be used to identify clinical settings in which laparoscopy may be beneficial rather than to justify its uniform application to all patients or operative strategies.

This study has several limitations. First, it focused on patients with diverticular disease who underwent surgery; thus, it did not address whether surgery is preferable to nonoperative management. Therefore, the findings should be interpreted as evidence regarding the choice of surgical approach among patients already selected for operative intervention rather than as evidence supporting surgery itself. Second, because no standardized risk‐of‐bias tool was applied, methodological quality was not formally graded within each study‐design category. Consequently, the consistency of favorable findings may have been overstated, particularly because several observational studies were susceptible to similar forms of selection bias and residual confounding. Findings from observational studies should therefore be interpreted as associations rather than evidence of a causal effect of the laparoscopic approach. Third, study design, disease severity, clinical setting, operative strategy, and outcome definitions varied among the included studies. Finally, data on surgeon experience, institutional expertise, conversion practices, and intraoperative outcomes such as ureteral injury and blood loss were not uniformly captured.

In conclusion, laparoscopy offers the clearest benefits in elective diverticular surgery, whereas its role in emergency surgery and perforated disease remains uncertain. The choice of approach should therefore be driven not by technique alone but by the interaction among disease severity, patient condition, operative strategy, and institutional expertise. This framework supports the selective use of laparoscopy, with careful patient selection, appropriate surgical planning, and timely conversion when necessary.

## Author Contributions

**Yudai Fukui:** investigation, data curation. **Koya Hida:** conceptualization, project administration, writing – review and editing. **Hiroya Takeuchi:** supervision, writing – review and editing. **Marie Hanaoka:** investigation, data curation, writing – original draft. **Yusuke Kinugasa:** writing – review and editing.

## Funding

The authors have nothing to report.

## Ethics Statement

Ethical approval and informed consent were not required because this study was a structured narrative review of previously published literature and did not involve the collection or analysis of individual patient data.

## Consent

The authors have nothing to report.

## Conflicts of Interest

Yusuke Kinugasa received speaker honoraria from Intuitive Surgical Inc., Johnson & Johnson KK, and Medtronic, Japan, and is an Associate Editor at *Annals of Gastroenterological Surgery*. The other authors declare no conflicts of interest.

## Supporting information


**Table S1:** Detailed matrix of perioperative outcomes comparing minimally invasive surgery with open surgery for diverticulitis.

## Data Availability

The data that support the findings of this study are available on request from the corresponding author. The data are not publicly available due to privacy or ethical restrictions.
